# Poorer patient-reported outcome and increased risk of revision at a 5-year follow-up among patients with septic arthritis following anterior cruciate ligament reconstruction: a register-based cohort study of 23,075 primary anterior cruciate ligament reconstructions

**DOI:** 10.1007/s00167-023-07498-6

**Published:** 2023-07-03

**Authors:** Jesper Kraus Schmitz, Osama Omar, Adam Nordkvist, Henrik Hedevik, Per-Mats Janarv, Anders Stålman

**Affiliations:** 1 Stockholm Sports Trauma Research Center, Department of Molecular Medicine and Surgery, Karolinska Institutet, Stockholm, Sweden; 2 Department of Clinical Science and Education, Södersjukhuset, Karolinska Institutet, Vo Ortopedi, Södersjukhuset, Sjukhusbacken 10, 11883 Stockholm, Sweden; 3 Department of Health, Medicine and Caring Sciences, Linköping University, Linköping, Sweden

**Keywords:** ACL, Osteoarthritis, Revision, PROMs

## Abstract

**Purpose:**

The primary aim of this study is to analyse the patient-reported outcomes after ACLR complicated by septic arthritis. The secondary aim is to examine the 5-year risk of revision surgery after primary ACLR complicated by septic arthritis. The hypothesis was that patients with septic arthritis after ACLR are more likely to have lower PROMs scores and an increased risk of revision, compared with patients without septic arthritis.

**Materials and methods:**

All primary ACLRs, with a hamstring or patellar tendon autograft (*n* = 23,075), in the Swedish Knee Ligament Register (SKLR) between 2006 and 2013 were linked with data from the Swedish National Board of Health and Welfare to identify patients with postoperative septic arthritis. These patients were verified in a nationwide medical records analysis and compared with patients without infection in the SKLR. The patient-reported outcome was measured using the Knee injury and Osteoarthritis Index Score (KOOS) and the European Quality of Life Five Dimensions Index (EQ-5D) at 1, 2 and 5 years postoperatively and the 5-year risk of revision surgery was calculated.

**Results:**

There were 268 events of septic arthritis (1.2%). The mean scores on the KOOS and EQ-5D index were significantly lower for patients with septic arthritis on all subscales on all follow-up occasions compared with patients without septic arthritis. Patients with septic arthritis had a revision rate of 8.2% compared with 4.2% in patients without septic arthritis (adjusted hazard ratio 2.04; confidence interval 1.34–3.12).

**Conclusion:**

Patients suffering from septic arthritis following ACLR are associated with poorer patient-reported outcomes at 1-, 2- and 5-year follow-ups compared with patients without septic arthritis. The risk of revision ACL reconstruction within 5 years of the primary operation for patients with septic arthritis following ACLR is almost twice as high, compared with patients without septic arthritis.

**Level of evidence:**

III.

## Introduction

Septic arthritis following anterior cruciate ligament reconstruction (ACLR) is a rare yet severe adverse event. It often requires multiple surgical interventions and long-term treatment with antibiotics and may lead to joint damage and permanent disability [23, 35]. In the literature, the incidence of septic arthritis following ACLR is reported as being between 0.28 and 1.1%, with identified risk factors such as male sex, longer operating time, graft choice and choice of perioperative antibiotic prophylaxis [3, 4, 14, 22]. The infected joint is vulnerable to several mechanisms that can damage the cartilage, such as bacterial toxins and the host’s inflammatory response [32]. In the ACL-reconstructed knee, there is also a risk of graft damage and, eventually, graft failure. In the acute management of septic arthritis, the rate of graft preservation has been estimated at 86% [30].

Patient-reported outcomes (PROs) following ACLR are often measured using the Knee injury and Osteoarthritis Outcome Score (KOOS) and the European Quality of Life Five Dimensions (EQ-5D) [8, 27]. Patient-reported outcome measurements (PROMs) following septic arthritis after ACLR have previously been studied in smaller cohorts ranging from 10 to 27 patients, with a mean follow-up time of 36–60 months [5, 31]. The results showed that some of the subscales were reported with inferior results in the septic arthritis group. However, the differences were small and sometimes non-existent [5, 31]. Due to the small populations, there is a lack of statistical power.

The general risk of revision ACLR is estimated to be between 2.6 and 7.7% [10, 11, 15]. The identified risk factors for revision ACLR include the choice of graft, age at the time of surgery and the level of activity at the time of index surgery [7, 10, 13, 20]. So far, the risk of revision ACLR due to septic arthritis in a larger cohort has not been well described in the literature.

The primary aim of this comprehensive nationwide study is to analyse PROMs after primary ACLR complicated by septic arthritis. The secondary aim is to examine the 5-year risk of revision surgery after primary ACLR complicated by septic arthritis. The hypothesis was that patients with septic arthritis after ACLR are more likely to have lower PROMs scores and an increased risk of revision, compared with patients without septic arthritis.

## Materials and methods

The study was approved by the regional ethics committee at Karolinska institute, Stockholm, Sweden 2013/1257-31/3 and 2017/408-32).

All primary ACLRs using a hamstring or patellar tendon autograft, registered in the Swedish Knee Ligament Register (SKLR) between 2006 and 2013, were included. Surgeries performed using other types of graft were excluded. The SKLR was established in 2005 and covers more than 90% of all ACLRs in Sweden. The register consists of two parts: perioperative notes by the surgeon, which include information such as associated injuries, associated procedures and the type and size of the graft used, and patient self-reported outcome data (KOOS and EQ-5D), recorded preoperatively and 1, 2, 5 and 10 years postoperatively.

Patients with septic arthritis were identified from a previous study, in which data on septic arthritis were obtained by combining information from registers at the National Board of Health and Welfare with data from medical records [14]. The included variables were extracted from the SKLR and consisted of sex, age at surgery, body mass index (BMI), smoking, in-/outpatient surgery, cartilage lesion, meniscal resection, meniscal suture, choice of graft, operating time and perioperative antibiotics. The variable of “perioperative antibiotics” is divided into four categories in the SKLR: cloxacillin, clindamycin, cefuroxime and other drugs. The two most used categories were selected for the analysis.

**Fig. 1:**
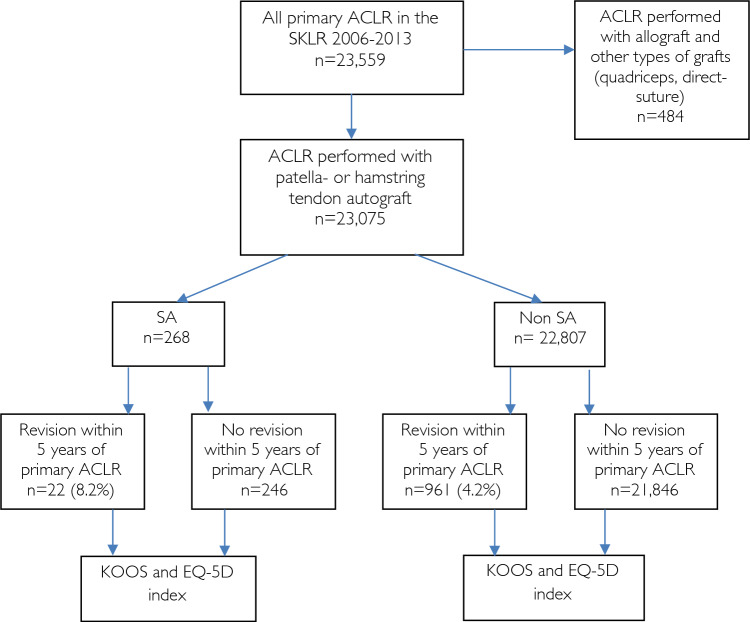
Flowchart of patient selection. ACLR, anterior cruciate ligament reconstruction; SKLR, Swedish Knee Ligament Register

PROMs data were obtained from the SKLR and included 1-, 2- and 5-year follow-ups of the five subscales on the KOOS (pain, symptoms, activities of daily living (ADL), sport and recreational function (Sport/Rec) and knee-related quality of life (QoL)), as well as the EQ-5D index. The thresholds for treatment failure and patient-acceptable symptom state (PASS), as described by Ingelsrud et al. and Muller et al. respectively, were applied to the KOOS subscales [12, 21]. The PROM data of patients who underwent revision ACLR within 5 years of the primary ACLR were included until the time of their revision surgery. Revision ACLR data were obtained from the SKLR. In the SKLR, reoperations are registered as revisions only if the ACL was reconstructed again after a primary ACLR. The risk of revision ACLR was analysed within 5 years from the primary ACLR (Fig. 1).

### Statistical analysis

Statistics, descriptive and analyses, were calculated using SPSS for MS Windows version 26.0 (IBM Corp., Armonk, New York). All the variables were summarized using standard descriptive statistics such as frequency, mean and standard deviation. Differences between groups in categorical variables were analysed using Pearson’s *χ*^2^ method. Differences between groups in continuous variables were analysed using Student’s t test for independent variables, provided that the distributions were not severely skewed. The data on the KOOS and EQ-5D were analysed using linear mixed models with a restricted maximum likelihood method to estimate change over time. Individuals with at least one PROM were included in the models. The within-subject variable time was entered as a repeated effect with an unstructured covariance structure. Time and the between-subject variable group and the interaction between time × group were entered as fixed effects in the model. The final models also included the covariates of age at surgery, sex, choice of graft and cartilage injury. Within-subject effects are presented at each time point with a 95% confidence interval (CI) and *p* value. Between-subject effects and mean change are presented at each time point with a 95% CI and *p* value.

Proportional Cox regression was used to estimate the risk of revision ACLR within 5 years of the primary ACLR. Revision within 5 years was entered as the status variable, years to revision as the time variable and septic arthritis as the prognostic factor in the model. The final models also included the covariates of age at surgery, sex, choice of graft and cartilage injury. Individuals were right censored if they survived up to 5 years, i.e. no reported event within the study period, or if they were reported dead before the end of the study. The proportional hazard assumption was checked both graphically and statistically, using a log-minus-log plot entering the variable of septic arthritis as a stratum, and by modelling the time × group interaction in a time-dependent Cox model. Non-parallel lines in the log-minus-log plot and a significant interaction effect in the model, indicating a time-dependent covariate, i.e. the proportional hazard assumption, was violated. The hazard ratio (HR) with a 95% CI is presented. The level of significance was 5% (two-tailed) in all analyses.

## Results

The cohort consisted of 23,075 patients who underwent a primary ACLR with a hamstring or patellar tendon autograft and 268 patients had septic arthritis (1.2%). Significant differences between the group with septic arthritis and the group without septic arthritis were found for the variables of sex, cartilage lesion, operating time, choice of graft, choice of perioperative antibiotics and revision ACLR within 5 years (Table 1).

**Table 1:** Descriptive data for the group with (*n* = 268) and without (*n* = 22,807) septic arthritis

		Septic arthritis		
		Yes	No	*p* value
*Patient demographics*				
Sex	Total *n*	23,075		
Women	*n* (%)	83 (31.0)	9708 (42.6)	< 0.001
Men	*n* (%)	185 (69.0)	13,099 (57.4)	
Age at surgery in years	Total *n*	23,075		
	M (Range)	27.1 (11–53)	26.8 (7–67)	n.s
Body mass index, BMI	Total *n*	11,927		
	M (SD)	25.1 (2.7)	24.6 (3.4)	n.s
Smoking	Total *n*	12,094		
Yes	*n* (%)	8 (5.9)	682 (5.7)	n.s
No	*n* (%)	127 (94.1)	11,277 (94.3)	
*Perioperative data*				
Type of surgery	Total *n*	23,075		
Outpatient surgery	*n* (%)	215 (80.2)	17,488 (76.7)	n.s
Inpatient surgery	*n* (%)	53 (19.8)	5319 (23.3)	
Cartilage lesion	Total *n*	23,075		
Yes	*n* (%)	87 (32.5)	5993 (26.3)	0.022
No	*n* (%)	181 (67.5)	16,814 (73.7)	
Meniscal resection	Total *n*	23,075		
Yes	*n* (%)	86 (32.1)	6999 (30.7)	n.s
No	*n* (%)	182 (67.9)	15,808 (69.3)	
Meniscal suture	Total *n*	23,075		
Yes	*n* (%)	20 (7.5)	1530 (6.7)	n.s
No	*n* (%)	248 (92.5)	21,277 (93.3)	
Choice of graft	Total *n*	23,075		
Hamstring autograft	*n* (%)	263 (98.1)	21,657 (95.0)	0.018
Patellar autograft	*n* (%)	5 (1.9)	1150 (5.0)	
Operating time in min	Total *n*	21,832		
	M (Range)	81.2 (35–246)	73.7 (17–304)	< 0.001
Perioperative antibiotics	Total *n*	22,538		
Cloxacillin	*n* (%)	249 (94.3)	21,741 (97.6)	0.001
Clindamycin	*n* (%)	15 (5.7)	533 (2.4)	
Revision surgery within 5 years	Total *n*	23,075		
Yes	*n* (%)	22 (8.2)	961 (4.2)	0.001
No	*n* (%)	246 (91.8)	21,846 (95.8)	

*M* mean, *SD* standard deviation, *n.s* non-significant

### Patient-reported outcomes

**Fig. 2:**
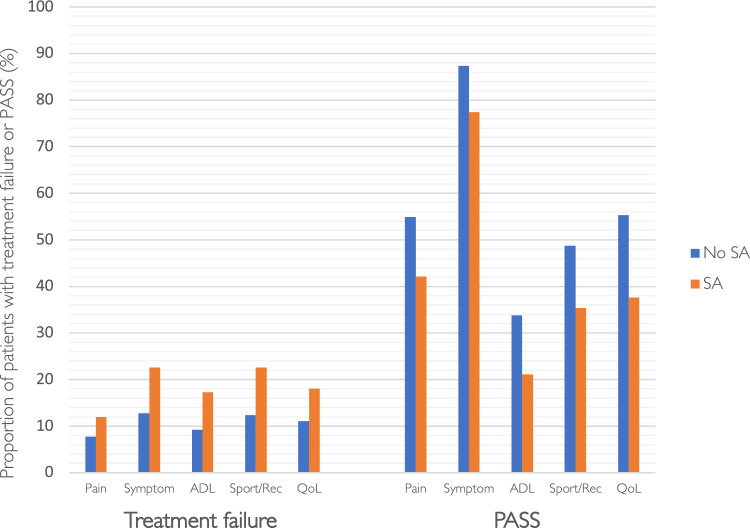
Proportion of patients with treatment failure and patient-acceptable symptom state (PASS) in the groups with and without septic arthritis (SA), displayed with the five KOOS subscales; ADL, activities of daily living; Sport/Rec, sport and recreational function; QoL, knee-related quality of life; 2 years postoperatively

Two years postoperatively, patients with septic arthritis had a larger proportion of treatment failure and fewer PASS on all KOOS subscales, compared with patients without septic arthritis (Fig. 2).

**Fig. 3:**
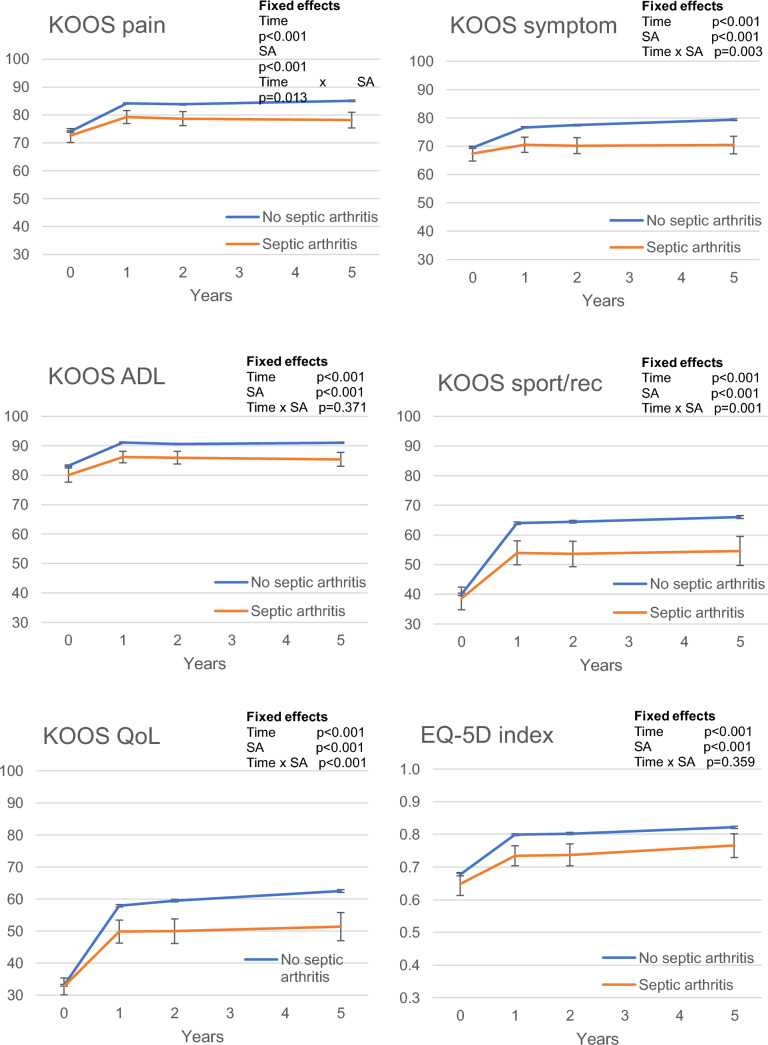
Mean values for the KOOS subscales and the EQ-5D index for the groups with and without septic arthritis preoperatively (0 years) and on each follow-up occasion (1, 2 and 5 years postoperatively). Adjusted for the covariates of age at surgery, sex, cartilage lesion and choice of graft. Whiskers displaying 95% confidence interval. SA, septic arthritis; ADL, activities of daily living; sport/rec, sport and recreational function; QoL, knee-related quality of life

Within-subject effects showed that patients without septic arthritis improved significantly on all subscales of the KOOS and EQ-5D index on all follow-up occasions. Patients with septic arthritis did not show a significant improvement on the KOOS subscale of symptoms, but they did show a significant improvement on all the other subscales and the EQ-5D index on all follow-up occasions (Fig. 3).

Between-subject effects of the KOOS subscale of ADL and the EQ-5D index showed a significant difference between patients with septic arthritis and those without septic arthritis preoperatively, with lower scores observed in the septic arthritis group. However, the mean changes of score on the follow-up occasions were not significant. All other KOOS subscales showed no significant difference between the groups preoperatively and patients without septic arthritis showed a significantly higher mean change of score on all follow-up occasions (Fig. 3). The fixed effects of time x septic arthritis were significant on the KOOS subscales of symptoms, pain, sport and recreational function and knee-related quality of life (Fig. 3).

### Revision ACL reconstructions

**Fig. 4:**
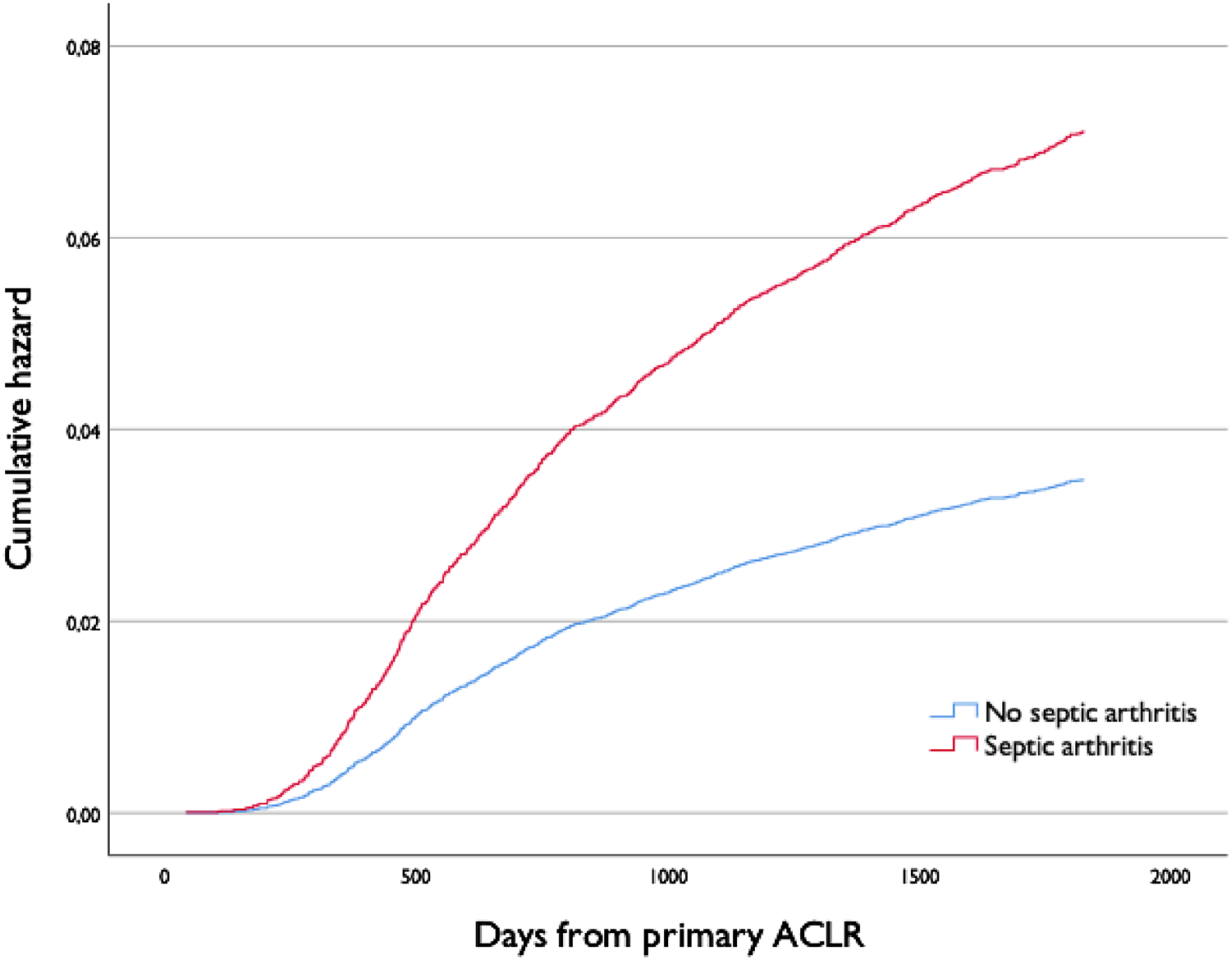
Adjusted, cumulative hazard rate for revision ACLR within 5 years of primary surgery

The overall rate of revision ACL reconstruction was 4.3%. Patients with septic arthritis had a significantly larger number of revision ACL reconstructions within 5 years of primary surgery, compared with patients without septic arthritis (8.2% vs 4.2%, *p* = 0.001) (Table 1). The adjusted risk of revision ACL reconstruction within 5 years of primary surgery was twice as high for patients with septic arthritis, compared with patients without septic arthritis (hazard rate ratio 2.04, 95% confidence interval (CI) 1.34–3.12) (Fig. 4). The median time from primary to revision ACL reconstruction was 800 days, with a range from 254 to 1825 days for patients with septic arthritis, and 1020 days, with a range from 42 to1825 days for patients without septic arthritis.

## Discussion

The most important finding of this study is that patients with septic arthritis had poorer PROs than those without septic arthritis, measured with the KOOS and EQ-5D index 1, 2 and 5 years postoperatively. Additionally, the 5-year risk of requiring a revision ACLR among patients with septic arthritis was found to be twice as high (HR 2.04) compared with patients without septic arthritis.

PROMs scores among patients with septic arthritis after ACLR compared with patients without septic arthritis were significantly lower across all the subscales on the KOOS and EQ-5D index at 1, 2 and 5 years of follow-up. There was a gradual improvement in KOOS scores following surgery on each follow-up occasion among patients without septic arthritis. A different trend was observed among patients with septic arthritis on the KOOS subscales of symptoms, pain, sport and recreational function and knee-related quality of life, where there was a tendency towards either similar or even poorer scores at the 2- and 5-year follow-ups compared with the 1-year follow-up. The same subscales show a significant improvement with time for the group without septic arthritis compared with the group with septic arthritis. Overall, this demonstrates that patients who develop postoperative septic arthritis have a poor long-term outcome.

There are several studies that have reported on PROs after an ACLR that was complicated by septic arthritis. However, they are all based on single institutions and present a small number of infected patients. In a review by Makhni et al. with a mean follow-up of 44 months, PROs were presented with Lysholm scores (15 of 19 studies, 160 patients) and showed that patients with septic arthritis had similar scores compared with patients without septic arthritis. The International Knee Documentation Committee scores (4 of 19 studies, 50 patients) were, however, lower among patients with septic arthritis [19].

Boström Windhamre et al. conducted a study on 27 patients with septic arthritis after ACLR and found no significant difference in the improvement in KOOS scores between patients with septic arthritis and the control group, during a mean follow-up time of 60 months [5]. The EQ-5D was included in their analysis, but, due to a small number of responses, the results were not presented. Similar results were reported by Abdel-Aziz et al., who conducted a study on 24 patients with septic arthritis after ACLR and reported no difference in KOOS scores at a mean follow-up of 59 months, compared with patients without septic arthritis [2]. In a cohort of 6,030 patients, Von Essen et al. showed that subsequent surgery after ACLR leads to an inferior outcome on the PASS and treatment failure at the two-year follow-up [36]. Only 5% of the subsequent surgeries in that cohort were due to septic arthritis.

In contrast to previous studies, this comprehensive nationwide study was able to report clear and highly significantly poorer PROs for patients with septic arthritis after ACLR, compared with patients without septic arthritis. After adjusting our PROMs data for sex, age at surgery, cartilage lesion and choice of graft, the differences between the group with septic arthritis and the group without septic arthritis persisted*.* The reasons for these lower scores have not yet been fully explained. It is thought that the damage to a septic joint is caused primarily by three mechanisms: direct injury to cartilage by bacterial enzymes and toxins, the release of collagen-degrading enzymes due to host inflammation and high intra-cellular pressure leading to joint asphyxia [28, 32]. Rötterud et al. have reported that full-thickness focal cartilage lesions were associated with lower postoperative KOOS scores [29]. It is therefore possible that the damaged cartilage itself could be one explanation of the lower PROMs scores. However, in the long term, it is possible that the infection may cause the more rapid development of osteoarthritis than would normally be expected [17]. Patients with postoperative osteoarthritis have obtained inferior results on the KOOS subscales of sport and recreational function and knee-related quality of life [17, 33].

Another possibility is the development of arthrofibrosis, which can occur after an infection and cause a poorer outcome [18].

The data of this study shows that the KOOS subscales of sport and recreational function, as well as knee-related quality of life, did not improve over time to the same extent as those in patients without septic arthritis, which could reflect a possible degenerative process in the knee joint.

The overall incidence of revision ACLR in our study was 4.3%. The incidence of revision ACLR was almost twice as high (8.2%) in the group of patients who developed postoperative septic arthritis after the primary ACLR, which is a novel finding that has not previously been reported.

Postoperative septic arthritis is treated with prompt arthroscopic irrigation and debridement in the operating room, together with appropriate intravenous antibiotics [37]. According to a review by Saper, the average number of irrigation and debridement procedures required for the successful treatment of the infection is 1.5 procedures [30]. The same review reported a success rate of 85.5% for graft preservation [30]. However, some authors recommend the removal of the graft at the first irrigation and debridement procedure, followed by antibiotic treatment and an early revision procedure [6]. In a recent study, it was found that 21 of 23 patients (91%) who underwent early hardware removal and graft preservation only required a single arthroscopic debridement without compromising the stability of the knee [16]. Comparable outcomes have been reported in patients treated with graft retention versus those treated with graft removal followed by an early revision ACLR (6–12 months after septic arthritis) [24]. In Sweden, the graft is normally preserved at the initial irrigation and debridement procedures [5]. The data of this study shows that patients with septic arthritis run an increased risk of revision ACLR and a poorer patient-reported outcome which could indicate that the graft is of poorer quality and probably entails an increased risk of graft failure.

Several risk factors have been identified for revision ACLR, including younger age, a hamstring autograft and a higher level of activity at the time of injury [7, 9, 10, 13, 20, 34]. In a review by Rahardja et al., conflicting evidence relating to the risk of revision ACLR was reported regarding patient sex and concomitant injuries such as meniscal and cartilage lesions [25]. After adjusting for the above-mentioned factors, septic arthritis is still a major risk factor for revision ACLR with an HR of 2.04.

This is a register-based study and it has several limitations. The number of irrigation and debridement procedures, and the rate of graft removal during treatment, is unknown. It is possible that patients who undergo graft removal during treatment may run a higher risk of requiring a revision ACLR. There is also a lack of information at follow-up such as return to sport and objective knee function. In addition, we do not have access to follow-up X-rays where the possible development of osteoarthritis could be analysed. Limitations regarding the cohort have been addressed in a previous article [14].

The KOOS and EQ-5D data have an increasing number of missing values with every follow-up and the response rate for the two-year follow-up data during the study period is 50% [1]. However, in a non-response analysis of the PROM data from 2012, Reinholdsson et al. concluded that the data in the SKLR are valid [26].

The main strength of this study is that it is the largest to date reporting on PROMs and the risk of revision ACLR for patients with septic arthritis following ACLR.

## Conclusion

Patients suffering from septic arthritis following an ACLR are associated with poorer patient-reported outcomes at one-, 2- and 5-year follow-ups compared with patients without septic arthritis.

The risk of revision surgery within 5 years of the primary operation for patients with septic arthritis following an ACLR is almost twice as high compared with patients without septic arthritis.

## Data Availability

Not applicable.
